# Human midbrain dopaminergic progenitors

**DOI:** 10.1111/cpr.13563

**Published:** 2023-10-25

**Authors:** Yu‐Kai Wang, Lin Feng, Ai‐Jin Ma, Jie Hao, Ying Zhang, Yue‐Jun Chen, Zhi‐Guo Chen, Jun‐Ying Yu, Yan Liu, Chang‐Mei Liu, Yu Zhang, Chang‐Lin Wang, Zhao‐Qian Teng, Jia‐Xi Zhou, Tian‐Qing Li, Liu Wang, Bo‐Qiang Fu, Yu Vincent Fu, Li‐Jun Zhu, Ling‐Min Liang, Jia‐Ni Cao, Lei Wang, Qi Zhou, Andy Peng Xiang, Bao‐Yang Hu, Tong‐Biao Zhao

**Affiliations:** ^1^ State Key Laboratory of Stem Cell and Reproductive Biology, Institute of Zoology Chinese Academy of Sciences Beijing China; ^2^ Beijing Institute for Stem Cell and Regenerative Medicine Beijing China; ^3^ Institute for Stem Cell and Regeneration Chinese Academy of Sciences Beijing China; ^4^ National Stem Cell Resource Center Chinese Academy of Sciences Beijing China; ^5^ University of Chinese Academy of Sciences Beijing China; ^6^ Chinese Society for Stem Cell Research Shanghai China; ^7^ Beijing Technology and Business University Beijing China; ^8^ Nuwacell Biotechnologies Co., Ltd., Hefei China; ^9^ Institute of Neuroscience, Key Laboratory of Neuroscience, CAS Center for Excellence in Brain Science and Intelligence Technology Chinese Academy of Sciences Shanghai China; ^10^ Shanghai Center for Brain Science and Brain‐Inspired Intelligence Technology Shanghai China; ^11^ Center of Neural Injury and Repair Beijing Institute for Brain Disorders Beijing China; ^12^ Cell Therapy Center, Beijing Institute of Geriatrics Xuanwu Hospital Capital Medical University, National Clinical Research Center for Geriatric Diseases, Key Laboratory of Neurodegenerative Diseases, Ministry of Education Beijing China; ^13^ Institute for Stem Cell and Neural Regeneration, State Key Laboratory of Reproductive Medicine, School of Pharmacy Nanjing Medical University Nanjing China; ^14^ Zephyrm Biotechnologies Co., Ltd., Beijing China; ^15^ China National Institute of Standardization Beijing China; ^16^ Institute of Hematology and Blood Diseases Hospital Chinese Academy of Medical Sciences & Peking Union Medical College Beijing China; ^17^ State Key Laboratory of Primate Biomedical Research, Institute of Primate Translational Medicine Kunming University of Science and Technology Kunming Yunnan China; ^18^ Yunnan Key Laboratory of Primate Biomedical Research Kunming Yunnan China; ^19^ National Institute of Metrology Beijing China; ^20^ State Key Laboratory of Microbial Resources Institute of Microbiology, Chinese Academy of Sciences Beijing China; ^21^ Institute of Scientific and Technical Information of China Beijing China; ^22^ Center for Stem Cell Biology and Tissue Engineering, Key Laboratory for Stem Cells and Tissue Engineering, Ministry of Education Sun Yat‐Sen University Guangzhou China

## Abstract

Human midbrain dopaminergic progenitors (mDAPs) are one of the most representative cell types in both basic research and clinical applications. However, there are still many challenges for the preparation and quality control of mDAPs, such as the lack of standards. Therefore, the establishment of critical quality attributes and technical specifications for mDAPs is largely needed. “Human midbrain dopaminergic progenitor” jointly drafted and agreed upon by experts from the Chinese Society for Stem Cell Research, is the first guideline for human mDAPs in China. This standard specifies the technical requirements, test methods, inspection rules, instructions for usage, labelling requirements, packaging requirements, storage requirements, transportation requirements and waste disposal requirements for human mDAPs, which is applicable to the quality control for human mDAPs. It was originally released by the China Society for Cell Biology on 30 August 2022. We hope that the publication of this guideline will facilitate the institutional establishment, acceptance and execution of proper protocols, and accelerate the international standardization of human mDAPs for clinical development and therapeutic applications.

## 1 SCOPE

This document specifies the technical requirements, test methods, test regulations, instructions for use, labelling, packaging, storage, transportation and waste disposal requirements for human midbrain dopaminergic progenitors (mDAPs).

This standard is applicable for the production and testing of human mDAPs.

## 2 NORMATIVE REFERENCES

The following content constitutes indispensable articles of this standard through normative reference. For dated references, only the edition cited applies. For undated references, only the latest edition (including all amendments) applies:

- Ws 213 Diagnosis of hepatitis C.
- Ws 273 Diagnosis for syphilis.
- Ws 293 Diagnosis for human immunodeficiency virus (HIV)/AIDS.
- Pharmacopoeia of the People's Republic of China (2020).
- National Guide to Clinical Laboratory Procedures.

## 3 TERMS AND DEFINITIONS

The following terms and definitions apply to this document.

### 3.1 Human midbrain dopaminergic progenitors

The neural progenitor cells that can differentiated into dopaminergic neurons with dopamine synthesis and release capacity.

Note: Human mDAPs include progenitors with the characteristics of A9 (substantia nigra pars compacta) and A10 (ventral tegmental area) subtypes.

### 3.2 Midbrain

The middle portion of the future brain in vertebrate neural tube that connects the diencephalon and the hindbrain.

Note: This region gives rise to superior and inferior colliculus, substantia nigra and red nucleus.

### 3.3 Dopamine

A type of neurotransmitter, the precursor of norepinephrine and epinephrine, a key neurotransmitter decreased in Parkinson's disease brain.

## 4 ABBREVIATIONS

The following abbreviations are applicable for this document:

EBV: Epstein‐–Barr virus

HBV: hepatitis B virus

HCV: hepatitis C virus

HCMV: human cytomegalovirus

HIV: human immunodeficiency virus

HTLV: human T‐lymphotropic virus

mDAP: midbrain dopaminergic progenitor

STR: short tandem repeats

TP: *Treponema pallidum*

## 5 TECHNICAL REQUIREMENTS

### 5.1 Raw materials and ancillary materials

1. The collection of raw materials shall be in accordance with the domestically legal and ethical requirements.
2. Valid informed consent shall be signed by the donor in writing. The content of informed consent shall include but not limited to the research purpose, potential research and clinical applications under appropriate conditions, feedback on unexpected discoveries, potential commercial value and other issues affiliated to the research. Mechanisms for protecting the personal data and privacy of donors shall be established.
3. Organizations conducting cell research and/or production shall establish and implement standards for cell donor evaluation, collection methods, transportation, handover and storage to ensure the safety of donors and cells. The source material shall be accompanied with detailed documentation on the acquisition methods and donor information, including but not limited to the donor's general information, past medical history and family history. The information of donor's blood type (e.g., ABO, Rh) and human leucocyte antigen alleles shall be documented as necessary.
4. The origin of cells shall be traceable by referring to the relevant informed consent and/or their genomic and functional analysis data.
5. Ancillary materials such as culture medium and growth factors shall meet the corresponding quality control requirements. The ancillary materials can be inspected and tested if necessary.
6. When using animal serum, they shall be free of animal‐derived viral contamination. Serum from animals in geographical regions with prion epidemics (e.g. bovine spongiform encephalopathy) shall be prohibited.
7. The donors shall be screened for HIV, HBV, HCV, HTLV, EBV, HCMV and TP, and the results shall be documented.

### 5.2 Primary quality attributes

#### 5.2.1 Cell morphology

mDAPs under adherent culture exhibit irregular shapes; the nucleus of mDAP is indistinguishable under a microscope.

#### 5.2.2 Chromosome karyotype

The karyotype shall be normal as 46, XX or 46, XY.

#### 5.2.3 Cell viability

The cell viability shall be ≥80% before cryopreservation, and ≥30% post‐thawing.

#### 5.2.4 Cell markers

The double‐positive rate of FOXA2 and LMX1A shall be ≥70.0%, and the double‐positive rate of LMX1A and EN1 shall be ≥15%.

#### 5.2.5 Functional characterization

mDAPs shall be able to further differentiate into dopaminergic neurons, which has the following characteristics:

Cell morphology: some neurons aggregate and shall have extended nerve fibres.

Cell markers: FOXA2‐positive rate shall be ≥50%, LMX1A‐positive rate shall be ≥50%, TH‐positive rate shall be ≥10%.

Dopamine release: Under KCl stimulation, released dopamine shall be ≥0.5 ng/mL/30 min/2–4 × 10^6^ cells.

#### 5.2.6 Microorganisms

Fungi, bacteria, mycoplasma, HIV, HBV, HCV, HTLV, EBV, HCMV and TP shall be negative.

### 5.3 Process control

#### 5.3.1 Differentiation

1. The original cells, equipment, culture systems and operation procedures used for cell differentiation shall be defined and documented. The standard operating procedure for cell differentiation shall be established for reproducibility.
2. The differentiated cells shall be clearly defined by characteristics, including but not limited to morphology and marker gene expression.

#### 5.3.2 Cryopreservation

1. Cryopreserved cells shall be recorded with the cell name, culture conditions, passage number, operator's name and freezing date, and so on.
2. The cryopreservation procedure shall follow the known principles of cell cryopreservation and shall be recorded.

#### 5.3.3 Thawing

1. The thawing process shall be as rapid as possible to ensure the cell viability and biological activity.
2. Cell information shall be labelled including but not limited to the cell name, passage number, culture conditions, operator's name or initials, thawing date and time.

#### 5.3.4 Cell STR identification

The STR signature of mDAPs shall be consistent with that of original donor cells.

## 6 TEST METHODS

### 6.1 Cell morphology

Observe the morphology of cells using an optical microscope.

### 6.2 Chromosome karyotype

The method in the *Pharmacopoeia of the People's Republic of China* (*2020*) shall be followed.

### 6.3 Cell viability

The method in Annex A shall be followed.

### 6.4 Cell markers

The method in Annex B or flow cytometry analysis method shall be followed.

### 6.5 Functional characterization

The method in Annex C shall be followed.

### 6.6 Microorganisms

#### 6.6.1 Fungi

The “1101 Sterility Inspection Method” in *Pharmacopoeia of the People's Republic of China* (*2020*) shall be followed.

#### 6.6.2 Bacteria

The “1101 Sterility Inspection Method” in *Pharmacopoeia of the People's Republic of China* (*2020*) shall be followed.

#### 6.6.3 Mycoplasma

The “3301 Mycoplasma Inspection Method” in *Pharmacopoeia of the People's Republic of China* (*2020*) shall be followed.

#### 6.6.4 Human immunodeficiency virus

The nucleic acid test method in WS 293 shall be followed.

#### 6.6.5 Hepatitis B virus

The nucleic acid test method in *National Guide to Clinical Laboratory Procedures* shall be followed.

#### 6.6.6 Hepatitis C virus

The nucleic acid test method in WS 213 shall be followed.

#### 6.6.7 Human T‐lymphotropic virus

The nucleic acid test method in *National Guide to Clinical Laboratory Procedures* shall be followed.

#### 6.6.8 Epstein–Barr virus

The nucleic acid test method in *National Guide to Clinical Laboratory Procedures* shall be followed.

#### 6.6.9 Human cytomegalovirus

The nucleic acid test method in *National Guide to Clinical Laboratory Procedures* shall be followed.

#### 6.6.10 Treponema pallidum

The nucleic acid test method in WS 273 shall be followed.

## 7 INSPECTION RULES

### 7.1 Sampling method

1. Cells produced from the same production cycle, same production line, same source, same passage and same method are considered to be the same batch.
2. Three smallest units of packaging shall be randomly sampled from the same batch.

### 7.2 Quality inspection and release

1. Each batch of products shall be subject to the qualify inspection before release, and inspection reports shall be attached.
2. The quality inspection items shall include all the attributes specified in 5.2.

### 7.3 Review inspection

Review inspection shall be performed by professional cytological testing institutions or laboratories as necessary.

### 7.4 Decision rules

1. Products that pass all requirements in 5.2 for the quality inspection for release are considered to be qualified. Products that fail to pass one or more requirements in 5.2 for the quality inspection for release are considered to be unqualified.
2. Products that pass all requirements in 5.2 for the quality review inspection are considered to be qualified. Products that fail to pass one or more requirements in 5.2 for the review inspection are considered to be unqualified.

## 8 INSTRUCTIONS FOR USAGE

The instructions for usage shall include, but not limited to:

- product name;
- passage number;
- cell number;
- production date;
- lot number;
- production organization;
- storage conditions;
- shipping conditions;
- contact information;
- operation manual;
- execution standard number;
- manufacturing address;
- postal code;
- matters that need attention.

Note: Provide endotoxin content according to user's requirement.

## 9 LABELS

The label shall include but not limited to:

- product name;
- cell number;
- lot number;
- production organization;
- production date.

## 10 PACKAGE, STORAGE, AND TRANSPORTATION

### 10.1 Package

Appropriate materials and containers shall be selected to ensure the maintenance of the primary quality attributes of mDAPs.

### 10.2 Storage

1. Use the cryoprotective reagents that do not affect cell quality attributes.
2. Products shall be stored at a temperature below −130°C.

### 10.3 Transportation

1. According to the requirements for the use of cells, select appropriate transport modes to ensure the biological characteristics, safety, stability and effectiveness of mDAPs.
2. Cell transport shall take the following factors into account, including but not limited to, cell characteristics, cell containers, transport routes, transport equipment, modes of transport, transport risks and safeguards.
3. The control of transport conditions shall include but not limited to, the transportation modes and conditions, routes, time, personnel, address and cell information.
4. Cryopreserved cells shall be transported in dry ice or below −130°C, while fresh cells should be transported at 2–8°C.

## 11 WASTE DISPOSAL

1. Wastes generated during human mDAPs production and testing shall follow the waste cell management documents, strictly implement the management standards and make detailed records.
2. Any disposal of unqualified cells, leftover cells for disposal or donations during the research and production of human mDAPs shall be conducted properly in accordance with appropriate legal and ethical requirements.

## AUTHOR CONTRIBUTIONS

Tong‐Biao Zhao, Bao‐Yang Hu and Andy Peng Xiang contributed to conception and design. Yu‐Kai Wang, Yue‐Jun Chen, Ying Zhang, Yan Liu, Zhi‐Guo Chen, Jun‐Ying Yu, Chang‐Mei Liu, Yu Zhang, Tian‐Qing Li and Lin Feng drafted and revised the manuscript. Ai‐Jin Ma, Jie Hao, Chang‐Lin Wang, Zhao‐Qian Teng, Jia‐Xi Zhou, Liu Wang, Qi Zhou, Bao‐Yang Fu, Yu Vincent Fu, Li‐Jun Zhu, Ling‐Min Liang, Jia‐Ni Cao and Lei Wang critically read and revised the manuscript. All authors contributed to the manuscript read and approved the final manuscript.

## FUNDING INFORMATION

This research is supported by the National Key Research and Development Program of China, grant/award numbers: 2019YFA0110800, 2019YFA0903800, 2018YFA0108400, 2018YFE0204400, 2021YFA1101600; National Natural Science Foundation of China, grant/award number: 31970821; Joint Funds of the National Natural Science Foundation of China, grant/award number: U21A20396; CAS Project for Young Scientists in Basic Research, grant/award number: YSBR‐041.

## CONFLICT OF INTEREST STATEMENT

The authors declare no conflicts of interest.

## A.1 Instruments

1. Microscope
2. Haemocytometer

## A.2 Reagents

Unless otherwise stated, all reagents used shall be analytical grade. The water used for testing shall be deionized water (refer to GB/T 6682).

1. Phosphate‐buffered saline: pH 7.4.
2. Trypan blue solution

## A.3 Procedure

1. Preparing cell suspension

Harvest and suspend the cells with appropriate volume of phosphate‐buffered saline (A.2).

2 Trypan blue staining

Evenly mix the Trypan blue solution (A.2) with the cell suspension (A.3) at a volume ratio of 1:1.

3 Cell counting

Put coverslips on each chamber of a clean haemocytometer (A.1). Transfer 10 μL trypan blue/cell suspension (A.3) to the edge of the coverslip, allowing the cell suspension to fully fill the chambers under the coverslip without over‐or underfill. Repeat with the second chamber. Stand for 30 s, count the stained cells and the total cells under microscope (A.1.1), respectively.

For the 16 × 25 counting chamber, use the four 1 mm^2^ medium squares at the top left, top right, bottom left and bottom right of the chamber (i.e. 100 small squares) for counting. For the 25 × 16 counting chamber, use the five 1 mm^2^ medium squares at the top left, top right, bottom left, bottom right and centre of the chamber (i.e. 80 small squares) for counting. When there are cells on the lines of the large square, only cells on the top line and left line of the large square can be counted (or alternatively only cells on the bottom line and right line).

## A.4 Calculation and analysis

Cell viability is calculated according to Equation (A.1):

(A.1)
$$S=\frac{M-D}{M}\times 100\%.$$

where *S* indicated cell viability, *M* is total cell number, *D* is stained cell number.

The viability of cells is the mean of two duplicate samples. Two independent cell viability tests shall be performed on the same sample. The mean value of two independent viability tests is recorded as the viability of cells.

## A.5 Accuracy

The absolute difference value between the two independent tests, under the same conditions, shall not exceed 10% of their arithmetic mean.

## B.1 Instruments

Confocal laser scanning microscope

## B.2 Reagents

4 Phosphate‐buffered saline (PBS)
5 4% Paraformaldehyde (PFA)
6 Triton‐X100
7 Bovine serum albumin (BSA)
8 Hoechst33342
9 Anti‐fluorescence quenching agent
10 Colourless nail polish or coverslip sealant
11 Antibodies
12 Prepare the following solutions according to the relative requirements for immunofluorescent staining: blocking/permeabilization solution and antibody dilution solution.

## B.3 Sample storage

The fixed samples, PBS, 4% PFA and Hoechst33342 shall be stored at 2–8°C. The BSA and anti‐fluorescence quenching agent shall be stored at or below −20°C. Triton‐X100 and colourless nail polish shall be stored at room temperature. Antibodies shall be stored according to the manufacturer's instructions.

## B.4 Besting protocol

### B.4.1. Sample preparation and fixation

Put the sterile cover slips in the bottom centre of the cell culture dish and inoculate the cells. When the cells grow to the proper density, discard the culture medium and fix the cells using 4% PFA at room temperature for 15–30 min. Wash the cell samples with PBS for three times.

### B.4.2. Permeabilization and blocking

Permeate and block cells with PBS containing 0.3% Triton X100 and 2% BSA at room temperature for 1–2 h.

### B.4.3. Antibody incubation

Incubate the cells with the diluted antibodies according to the manufacturer's instructions.

### B.4.4. Washing

Wash the cells with PBS for three times (5–10 min each time).

### B.4.5. Nuclei staining

Discard PBS, cells are treated with Hoechst33342 solution (diluted 1:1000 in PBS) for 15 min.

### B.4.6. Coverslips sealing

Add 5 μL of anti‐fluorescence quenching agent to each cover slip, place the cover slip on a microscope slide carefully without forming air bubbles. Seal the edges of coverslips with colourless nail polish or coverslip sealant.

### B.4.7. Imaging

Choose at least three different visual fields randomly for imaging.

### B.4.8. Cell counting

Count the Hoechst positive cells in all images, at least 500 cells shall be counted in each image, and the total number shall not be less than 1500 cells.

The percentage of cell marker positive cells is calculated according to Equation (B.1):

(B.1)
$$Q=\frac{N}{M}\times 100\%$$

where *Q* is percentage of cell marker positive cells, *N* is antibody positive cells and *M* is Hoechst positive cells, ≥1500.

## C.1.1. Instruments

1. High‐performance liquid chromatography (HPLC) system.

## C.2 Reagents

1. Hanks' balanced salt solution (HBSS).
2. 56 mM potassium chloride (KCl) solution.
3. Dopamine standards.

## C.3 Experimental group

1. Control group: 56 mM KCl solution.
2. Test group: cells are treated with 56 mM KCl solution at 37°C for 30 min.

## C.4 Test protocol

### C.4.1. Sample preparation

Collect the differentiated cells and inoculate them into culture dishes after counting.

### C.4.2. Sample handling

After the cells attach to the surface of culture dishes, discard the supernatant. Cells are washed once with HBSS, and are treated with 56 mM KCl solution at 37°C for 30 min.

### C.4.3. Sample collection and storage

Collect the supernatant, store the samples in refrigerator at −80°C or ship samples with dry ice. Keep away from light to prevent the breakdown of dopamine.

### C.4.4. Sample testing

The peak areas of dopamine standards with concentration gradient, control group and test group were detected by HPLC.

### C.4.5. Analysis of results

The relationship between the peak area and the dopamine concentration of the sample is calculated according to Equation (C1):

(C1)
y=kx+b

where *y* is peak area of dopamine standards, *k* is the conversion factor, *x* is concentration of dopamine standards and *b* is constant.

The dopamine release is calculated according to Equation (C2):

(C2)
$$R=4\times 10^{6}\frac{X}{\mathrm{vn}}.$$

where *R* is dopamine release, *X* is dopamine concentration of cell samples, *v* is volume of KCl solution used to treat cell samples and *n* is total number of treated cells.

